# Selenoprotein W Ameliorates Experimental Colitis and Promotes Intestinal Epithelial Repair

**DOI:** 10.3390/antiox12040850

**Published:** 2023-04-01

**Authors:** Shaneice K. Nettleford, Chang Liao, Sarah P. Short, Randall M. Rossi, Vishal Singh, K. Sandeep Prabhu

**Affiliations:** 1 Department of Veterinary and Biomedical Sciences, The Pennsylvania State University, University Park, PA 16802, USA; 2 Department of Medicine-Infectious Diseases, University of California, San Francisco, CA 94143, USA; 3 Department of Medicine, Department of Gastroenterology, Hepatology, and Nutrition, Vanderbilt University Medical Center, Nashville, TN 37232, USA; 4 Mouse Transgenic Core Facility, Huck Institute of the Life Sciences, The Pennsylvania State University, University Park, PA 16802, USA; 5 Department of Nutritional Sciences, The Pennsylvania State University, University Park, PA 16802, USA

**Keywords:** inflammatory bowel disease, inflammation, yes-associated protein 1, epidermal growth factor receptor

## Abstract

Selenoprotein W (Selenow) is a ~9 kDa selenoprotein suggested to play a beneficial role in resolving inflammation. However, the underlying mechanisms are poorly understood. *SELENOW* expression in the human GI tract using ScRNAseq Gut Cell Atlas and Gene Expression Omnibus (GEO) databases revealed its expression in the small intestine and colonic epithelial, endothelial, mesenchymal, and stem cells and correlated with a protective effect in ulcerative colitis patients. *Selenow* KO mice treated with 4% dextran sodium sulfate (DSS) showed exacerbated acute colitis, with greater weight loss, shorter colons, and increased fecal occult blood compared to the WT counterparts. *Selenow* KO mice expressed higher colonic Tnfα, increased Tnfα^+^ macrophages in the colonic lamina propria, and exhibited loss in epithelial barrier integrity and decreased zonula occludens 1 (Zo-1) expression following DSS treatment. Expression of epithelial cellular adhesion marker (EpCam), yes-associated protein 1 (Yap1), and epidermal growth factor receptor (Egfr) were decreased along with CD24lo cycling epithelial cells in *Selenow* KO mice. Colonic lysates and organoids confirmed a crosstalk between Egfr and Yap1 that was regulated by Selenow. Overall, our findings suggest Selenow expression is key for efficient resolution of inflammation in experimental colitis that is mediated through the regulation of Egfr and Yap1.

## 1. Introduction

Inflammatory bowel disease (IBD), including Crohn’s disease (CD) and ulcerative colitis (UC), is a disease of global concern [1,2,3,4] characterized by remissions and relapses of symptoms such as diarrhea, weight loss, abdominal pain, and rectal bleeding [5]. The etiology of IBD is multifaceted, but evidence suggests that genetics as well as environmental factors are implicated in its pathogenesis [6]. Current treatments are geared towards promoting remission and improving the quality of life, but are also associated with undesired side effects, and may be ineffective in some patients [4,5,7,8]. Thus, there is an unmet need for novel therapies that are anti-inflammatory, lack side effects, facilitate resolution of inflammation, and promote intestinal regeneration.

The intestinal epithelium, comprised of goblet cells, tuft cells, and stem cells (located in the crypt base), and connected by tight junction proteins, is exposed to luminal antigens, the microbiota, and toxins, highlighting the importance of a protective barrier for the maintenance of gut homeostasis [9,10,11]. Disruption of the epithelial barrier integrity arises due to compromised tight junction proteins and epithelial cell death resulting from the activation and recruitment of immune cells, including macrophages that produce TNFα, a key cytokine implicated in inflammation and severity of IBD [9,11,12,13]. Thus, healing and repair of the epithelium may serve as a target for new therapies, where epidermal growth factor (Egf) receptor (Egfr) signaling is a key component [7,14]. Egfr is highly expressed in the epithelial crypt, especially the columnar base crypts (CBCs) [15,16,17]. Activation of Egfr results in proliferation, survival, and regeneration [7,18]. Yes-associated protein-1 (Yap1), the main effector of the Hippo pathway, modulates transcription of several genes involved in the repair of the epithelium [19,20]. In its inactive form, Yap1 is phosphorylated by large tumor suppressor 1 and 2 (Lats1/2), where it is sequestered in the cytoplasm by 14-3-3 or degraded by β-trcp, a E3 ubiquitin ligase [19]. Upon activation, Yap1 translocates to the nucleus and binds to a co-activator, TEAD, to activate transcription of target genes [21]. In head and neck squamous cell carcinoma cells, Egfr regulates the activation of Yap1 via Mob1 phosphorylation and inactivation [22]. Furthermore, Yap1 can regulate Egfr signaling through the Egfr ligand amphiregulin (Areg) that is also a target of Yap1 [23,24], suggesting a crosstalk between these pathways may positively impact epithelial regeneration.

The essential trace element selenium (Se) exists as the 21st amino acid, selenocysteine (Sec), in 25 (human) or 24 (mouse) selenoproteins via its unique ^Sec^tRNA encoded by Trsp [25]. Previous studies reported a decrease in Se levels in CD and UC patients, where the risk of UC was reduced with higher levels of Se, while preclinical studies demonstrated that supplementation with Se ameliorated dextran sodium sulfate (DSS)-induced colitis, where selenoproteins mediated the effect [26,27,28,29,30,31]. Selenoprotein W (Selenow) is a ~9 kDa selenoprotein expressed in muscle, brain, spleen, and colonic tissue in response to exogenous Se supply [32,33], though cellular identities, especially in the human intestinal epithelia are unclear. Using quantitative proteomics, we previously demonstrated that Selenow was a highly expressed selenoprotein in macrophages [34]. Selenow has thiol and peroxide reductase activity, interacts with 14-3-3 proteins and is known to regulate the binding of 14-3-3 to its partners CDC25B and Rictor [35,36,37]. Furthermore, Selenow was implicated in cell cycle progression of breast and prostate epithelial cells and in the proliferation of CD4^+^ T-cells [38,39]. Interestingly, Selenow regulated the activation and degradation of Egfr through suppression of its ubiquitination [40]. However, the pro-resolutory impact of Selenow expression in macrophages and epithelial cells in IBD needs to be further addressed.

As such, our aim was to understand the impact of the lack of Selenow on inflammation and regeneration and the mechanisms involved. We hypothesized that Selenow ameliorates DSS-induced colitis by suppressing inflammation and promoting epithelial regeneration through mechanisms involving Yap1 and Egfr signaling. Here we report that lack of Selenow results in increased pathology characterized by increased weight loss and shorter colons in mice treated with DSS. Our studies also indicate that the *Selenow* KO mice have a higher percentage of Tnfα producing macrophages compared to their WT counterparts. Furthermore, Selenow KO mice experience a greater disruption of the epithelial barrier as seen through functional and other biochemical assays. Using colonic lysates and organoids our studies indicate that Selenow activates Egfr, in addition to regulating the activation of Yap1 via its interaction with 14-3-3. Altogether, our studies show that Selenow inhibits gut inflammation and mediates epithelial repair via Yap1 and Egfr, thus underscoring the potential of Se supplementation as an adjunct therapy for IBD.

## 2. Materials and Methods

### 2.1. Mice and Dextran Sodium Sulfate-Induced Colitis

*Selenow* knock out (KO) mice on C57BL/6 background were generated at the Transgenic Core Facility of the Huck Institutes of the Life Sciences, The Pennsylvania State University. Briefly, using the CRISPR/Cas9 system, a whole body *Selenow* KO murine model was created by microinjection of sgRNA sequence, 5′-CTTCAAAGAACCCGGTGACC-3′ in complex with recombinant Cas9 (New England Biolabs, Ipswich, MA, USA) into single cell embryos. These were implanted into recipient mice and the germline transmission was followed in the subsequent progeny with PCR and surveyor assay and followed by DNA sequence confirmation. The loss of Selenow expression in homozygous *Selenow* KO mice was confirmed using immunoblotting PBMCs and ear tissue. Twelve- to fifteen-week-old male *Selenow* KO mice (*n* is between 4 and 24 mice depending on the experiment) and wild-type (WT) control mice (*n* is between 3 and 18 depending on the experiment) were administered 4% dextran sodium sulfate (DSS; *w*/*v*) in drinking water for 5 days, followed by 3 days of regular water to generate acute colitis. Weight changes and colon lengths were used as a measure of the severity of colitis. All procedures were pre-approved by the Institutional Animal Care and Use Committee at The Pennsylvania State University.

### 2.2. FITC Dextran Assay

The FITC dextran assay was conducted as previously described [41,42]. Briefly, food and water were removed from mice for 4 h followed by oral gavage with 500 mg/kg FITC dextran (Sigma Aldrich, St. Louis, MO, USA) reconstituted in sterile PBS. Mice were euthanized and blood collected via cardiac puncture. The FITC dextran levels in the serum were measured using a fluorimeter (Agilent BioTek Synergy LX, Santa Clara, CA, USA), and the concentration was calculated using an FITC standard curve.

### 2.3. Western Immunoblot

Total proteins were isolated from the proximal portion of the colon of mice using mammalian protein extraction reagent (MPER; ThermoFisher, Waltham, MA, USA). Thirty micrograms of lysate were subjected to SDS-PAGE (7% or 15%) gels to separate candidate proteins for western immunoblot analyses and processed as described earlier [41]. ImageJ (National Institutes of Health) was utilized to densitometrically evaluate the immunoreactive bands. All data provided were normalized to house-keeping controls in biological replicates. For co-immunoprecipitation experiments, total proteins were isolated from the proximal colon using radioimmunoprecipitation assay (RIPA; ThermoFisher, Waltham, MA, USA) buffer. Then 300–500 μg of protein was incubated with 0.5 μg of antibody for 1 h (Selenow pull down) or 6 h (14-3-3, Mob1A/B, and Yap1 pull down), followed by overnight incubation with Protein A/G Plus-Agarose beads (Santa Cruz Biotechnology, Dallas, TX, USA). Washed agarose beads were subjected to SDS-PAGE gel separation followed by immunoblotting, as above. Antibody dilutions were as follows: Selenow 1:1000 (Rockland Immunochemicals, Pottstown, PA, USA), pro-Il-1β 1:10,000 (GenTex Inc, Irvine, CA, USA), Il-1β 1:10,000 (GenTex Inc, Irvine, CA, USA), Tnfα 1:500 (Bioss Antibodies, Woburn, MA, USA), Egfr 1:1000 (ABClonal Technology, Woburn, WA, USA), pEGFR (Y1068) 1:1000 (ABClonal Technology, Woburn, WA, USA), Zo-1 1:500 (Proteintech), Yap 1:500 (ABClonal Technology, Woburn, WA, USA), pYap (S127) 1:500 (ABClonal Technology, Woburn, WA, USA), 14-3-3 1:1000 (ABClonal Technology, Woburn, WA, USA), Ubiquitin 1:1000 (Cell Signaling Technology, Danvers, MA, USA), phospho-tyrosine 1:4000 (Cell Signaling Technology, Danvers, MA, USA), β-Actin 1:20,000 (Fitzgerald Industries, Acton, MA, USA), Vinculin 1:5000 (Proteintech, Rosemont, IL, USA).

### 2.4. Gut Cell Atlas scRNA-Seq and GEO Database Query

The expression of *SELENOW* was queried using the Gut Cell Atlas scRNA-seq data on https://www.gutcellatlas.org (accessed on 30 March 2023) [43]. Data were selected to include the expression of *SELENOW* in the colonic and small intestine samples of healthy adults (both males and females) between the ages of 20–75. Data were mined from the Gene Expression Omnibus (GEO) database to deduce the expression of *SELENOW* in ulcerative colitis patients (https://www.ncbi.nlm.nih.gov/sites/GDSbrowser?acc=GDS3119; https://www.ncbi.nlm.nih.gov/geoprofiles/49193921) (accessed on 30 March 2023) [44].

### 2.5. Real Time PCR

Tri Reagent was used to isolate total RNA from colonic samples and real time PCR was conducted using the cDNA generated. SYBR primers were used to deduce the expression of Yap1 (forward primer: 5′-CGGCAGTCCTCCTTTGAGAT-3′; reverse primer: 5′-TCAGTTGCGAAAGCATGGC-3′ [45]), Areg (forward primer: 5′-AGGGGACTACGACTACTCAG-3′; reverse primer: 5′-GAAACTTGGCAGTGCATGGA-3′ [46]) and Gapdh (forward primer: 5′-TGACATCAAGAAGGTGGTGAAGC-3′; reverse primer: 5′-CCCTGTTGCTGTAGCCGTATTC-3′). The data are reported as 2^−∆CCT^.

### 2.6. Isolation of Colonic Lamina Propria Cells

The colonic lamina propria cells were isolated as described previously [41,47]. Briefly, colons were removed, cleaned, opened longitudinally, and cut into pieces (~5 mm). The colonic pieces were incubated in Hank’s buffered salt solution containing 0.25 M EDTA, 1 M HEPES, 10% FBS, and 1% penicillin/streptomycin, for 30 min at 37 °C with gentle shaking. The pieces were minced and digested using collagenase type I (1 mg/mL) and DNase I (10 μg/mL) in RPMI containing 5% FBS at 37 °C for 1 h. Following digestion, a Percoll gradient of 40–80% was used to recover the cells of the lamina propria, followed by centrifugation at 800× *g* for 20 min at room temperature.

### 2.7. Isolation of Intestinal Epithelial Crypts

The intestinal epithelial crypts were isolated using two different protocols. To examine the expression of Selenow in colonic crypts, cleaned colons were cut into sections and washed repeatedly (16 times) with cold PBS by shaking manually followed by incubation in Gentle Cell Dissociation Reagent (STEMCELL Technologies, Vancouver, BC, Canada) for 20 min at room temperature. Crypt fractions (1–5) were generated by shaking colonic pieces in PBS containing 0.1% BSA, where fractions 1 through 5 corresponded to cells that were progressively less differentiated. To generate crypts for flow cytometry and for organoid preparation, crypts were isolated as previously described [48,49,50]. Briefly, cleaned and cut colonic pieces were placed on a horizontal rocker, in PBS, for 15 min at 4 °C, washed twice in PBS, followed by incubation in PBS containing 2 mM EDTA on a horizontal rocker for 90 min at 4 °C. The samples were then subjected to gentle shaking in PBS containing 43.3 mM sucrose and 54.9 mM sorbitol for 1–2 min, until the crypts were released. Isolated crypts were used to generate organoids or further processed into single cell suspensions using TryPLE Express (Invitrogen, Waltham, MA, USA). For single cell suspensions, the crypts were pelleted, resuspended in 2 mL of TryPLE Express, and incubated in a 37 °C water bath for 10 min (with gentle shaking every 3 min). After incubation, the samples were placed on ice for 1 min, followed by addition of PBS containing 3% FBS (flow buffer) and centrifugation at 500× *g* for 7 min. The samples were resuspended in flow buffer, passed through a 70 μm strainer, and subjected to flow cytometric analysis.

### 2.8. Flow Cytometry

The isolated colonic lamina propria and intestinal epithelial cells were stained with protein markers used to delineate macrophages and intestinal epithelial cells, respectively. Cells were stained with cell surface markers for 30 min in flow buffer, followed by fixing with 4% paraformaldehyde and permeabilization for intracellular staining. Macrophages producing Tnfα were marked as CD45^+^F480^+^CD11b^+^Tnfα^+^, while resident macrophages expressing Cx3Cr1 producing Tnfα were marked as CD45^+^F4/80^+^CD11b^+^Cx3cr1^+^Tnfα^+^. Epithelial cells were marked as EpCam^+^, while EpCam^+^CD24^lo^ was used to characterize cycling epithelial cells. Antibody dilutions were as follows: CD45 1:100 (Alexa Fluor 700; BioLegend, San Diego, CA, USA), F4/80 1:50 (APC; Miltenyi Biotec, Bergisch Gladbach, Germany), CD11b 1:100 (Fitc; BioLegend, San Diego, CA, USA), Cx3Cr1 1:100 (PE, BioLegend, San Diego, CA, USA) Tnfα 1:50 (Pe-Cy7; BD Biosciences, San Jose, CA, USA), EpCam 1:100 (Fitc; BioLegend, San Diego, CA, USA), CD24 1:100 (PE; BioLegend, San Diego, CA, USA).

### 2.9. Organoid Generation

Isolated intestinal epithelial crypts described above were used to generate organoids using the protocol by STEMCELL Technologies, with some modifications. Briefly, 200–250 colonic crypts were resuspended in Matrigel-intesticult media (1:1 ratio), where 50 μL was plated in each well of a 24 well plate. The plate was placed in an incubator at 37 °C for 10 min, after which, 700 μL of intesticult media was added to each well. The colonic organoids (colonoids) were cultured for a total of nine days and the media changed every 2–3 days. The organoids were treated with Gefitinib (Egfr inhibitor, 5 μM, Sigma Aldrich, Waltham, MA, USA), Verteporfin (Yap1 inhibitor, 1 μM, Sigma Aldrich, Waltham, MA, USA), or Vehicle for 2 days starting at day 7. Organoids were imaged on day 7 and the area and perimeter of the organoids were calculated using the ImageJ program (National Institutes of Health).

### 2.10. Immunofluorescence

The distal portion of the colons from WT and *Selenow* KO mice were stored in 10% (*v*/*v*) buffered formalin, followed by sectioning at the Histopathology Core Facility, Animal Diagnostics Laboratory, Department of Veterinary and Biomedical Sciences, The Pennsylvania State University. To detect proteins of interest, the samples were deparaffinized, rehydrated, subjected to antigen retrieval, and blocked in PBS containing goat serum and 0.3% Triton X for 2 h. The sections were incubated in primary antibody overnight at 4 °C in the dark, washed in PBS, and incubated in appropriate secondary antibody for 1 h at room temperature. The sections were washed in PBS, mounted with anti-fading agent, VectaShield^®^ (Vector Laboratories, Newark, CA, USA), and imaged using the Leica DMi8 microscope (Leica Microsystems, Wetzlar, Germany) and LAS X software program. Ki67^+^ cells were enumerated by counting the number of Ki67^+^ cells in well-defined crypts. Organoids were harvested from matrigel and immunolabeled as described by Dekkers et al. [51]. Briefly, organoids were removed from the matrigel by incubation in cell recovery media (Corning Inc, Somerville, MA, USA) for 1 h at 4 °C, with rocking, followed by centrifugation at 70× *g* for 3 min. The organoids were then fixed in 4% (*v*/*v*) PFA for 45 min, incubated in Tween in PBS (0.1% *v*/*v*) for 10 min at 4 °C, and collected by centrifugation at 70× *g* for 5 min. These organoids were transferred to a 24 well plate, blocked in PBS containing 0.2% BSA and 0.1% Triton X for 15 min, and incubated with primary antibodies overnight at 4 °C. Three times washed organoids were incubated in secondary antibody overnight and transferred to a 1.5 mL tube, cleared in fructose–glycerol clearing solution for 20 min, mounted on slides, and imaged using the Leica DMi8 microscope (Leica Microsystems, Wetzlar, Germany) and LAS X software program.

### 2.11. Data Analysis

The data were graphed and analyzed using GraphPad Prism (version 9.0) and reported as standard error of the mean (±SEM). Either an unpaired nonparametric Mann–Whitney test, one-way ANOVA, or a two-way ANOVA was used to calculate statistical significance, where significance was considered when *p* < 0.05.

## 3. Results

### 3.1. Loss of Selenow Exacerbates DSS-Induced Colitis

**Figure 1:**
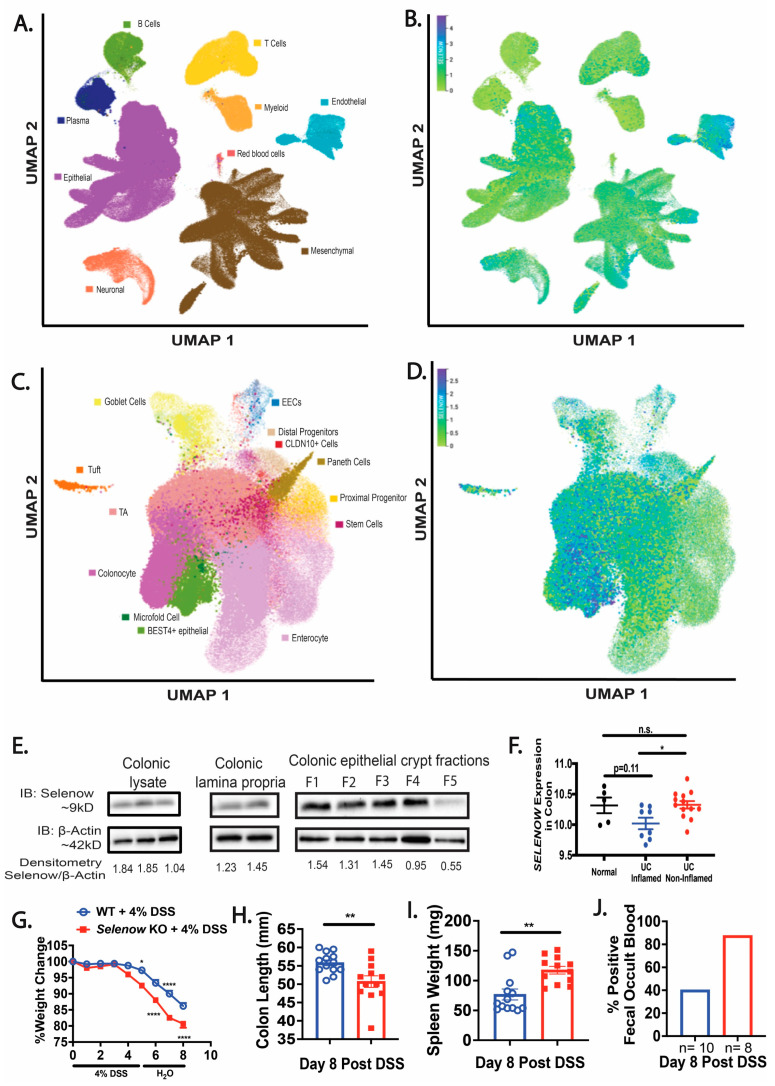
Critical role of Selenow in alleviating DSS-induced colitis. (**A**,**B**) *SELENOW* expression in the colonic and small intestinal cells from healthy human samples using the Gut Cell Atlas scRNA-seq data. (**C**,**D**) *SELENOW* expression in various cell types of healthy human colonic and small intestinal epithelium from the Gut Cell Atlas scRNA-seq data. (**E**) Expression of Selenow in colonic lysates, colonic lamina propria, and colonic epithelial crypts. (**F**) *SELENOW* expression in the colon of UC patients. (**G**) Percent weight change of WT and *Selenow* KO mice following administration of 4% DSS (WT = 17 mice; *Selenow* KO = 15). (**H**) Colon length of WT and *Selenow* KO mice at day 8 post DSS (WT = 13 mice; *Selenow* KO = 12). (**I**) Splenic weight of WT and *Selenow* KO mice at day 8 post DSS (WT = 13 mice; *Selenow* KO = 12). (**J**) Percentage of fecal occult blood positive WT and *Selenow* KO mice at day 8 post DSS. Data indicate mean ± SEM. (**F**) One-way ANOVA (**G**) Two-way ANOVA (**H**,**I**) Unpaired non-parametric Mann–Whitney test. * *p* < 0.05, ** *p* < 0.01, **** *p* < 0.0001, n.s., not significant.

The Gut Cell Atlas was queried to understand the expression of *SELENOW* in human intestinal cells. Querying of the scRNA-Seq Gut Cell Atlas revealed that *SELENOW* is expressed in numerous cell types including the epithelial, endothelial, and mesenchymal cells of the human colon and small intestine (Figure 1A,B). Furthermore, our analysis also indicated *SELENOW* expression in different cells of the intestinal epithelium including colonocytes, goblet cells, and stem cells (Figure 1C,D). Western immunoblotting analysis indicated that Selenow was expressed in the colon, colonic lamina propria cells, and various fractions (F1–F5) of the colonic epithelial crypts in the mouse colon (Figure 1E). Interestingly, data mining of the GEO database revealed that *SELENOW* was decreased in the inflamed colons of UC patients compared to the non-inflamed colons of UC patients (Figure 1F). We found that *Selenow* KO mice administered DSS had a greater weight loss compared to their WT counterparts (Figure 1G). During the resolution phase (day 8) following DSS treatment, colons in *Selenow* KO mice were shorter and the spleens were heavier, when compared to the WT mice (Figure 1H,I). Furthermore, 87.5% (7 of 8 mice) of *Selenow* KO mice tested positive for fecal occult blood (FOB) when compared to 40% (4 of 10 mice) of the WT mice treated with DSS (Figure 1J). Taken together, these results suggest that the colonic expression of Selenow plays an important role in the protection from the symptoms of colitis and resolution following GI inflammation.

### 3.2. Higher Inflammation in Selenow KO Mice

**Figure 2:**
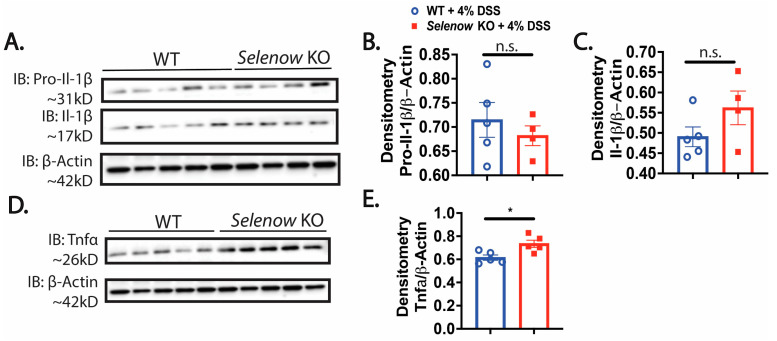
*Selenow* KO mice have higher colonic inflammation. (**A**) Representative immunoblot of pro-I1-1β and Il-1β, and (**B**,**C**) quantification of pro-I1-1β and Il-1β from colonic lysates of WT and *Selenow* KO mice at day 8 post DSS (WT = 5 mice; *Selenow* KO = 4 mice). (**D**) Representative immunoblot of Tnfα, and (**E**) quantification of Tnfα from colonic lysates of WT and *Selenow* KO mice at day 8 post DSS (WT = 5 mice; *Selenow* KO = 5 mice). Data indicate mean ± SEM. (**B**,**C**,**E**) Unpaired non-parametric Mann–Whitney test. * *p* < 0.05, n.s., not significant.

**Figure 3:**
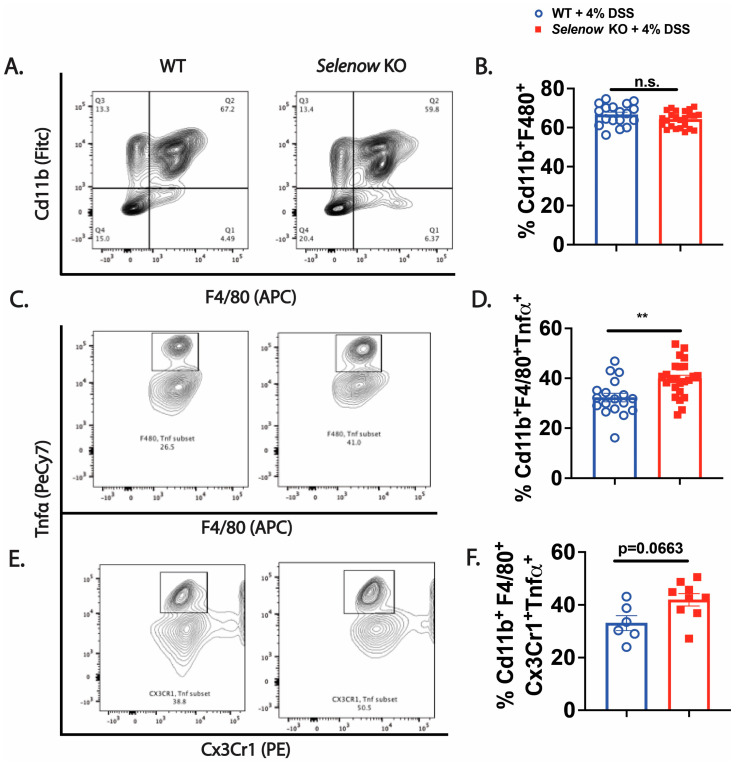
*Selenow* KO mice have a higher percentage of Tnfα producing macrophages in colonic lamina propria. (**A**) Representative flow plot and (**B**) percentage of macrophages (CD45^+^CD11b^+^F4/80^+^) isolated from cLP of WT and *Selenow* KO mice at day 8 post DSS (WT = 18 mice; *Selenow* KO = 24). (**C**) Representative flow plot and (**D**) percentage of macrophages (CD45^+^CD11b^+^F4/80^+^) producing Tnfα isolated from cLP of WT and *Selenow* KO mice at day 8 post DSS (WT = 18 mice; *Selenow* KO = 24). (**E**) Representative flow plot and (**F**) percentage of Cx3Cr1 macrophages (CD45^+^CD11b^+^F4/80^+^Cx3Cr1^+^) producing Tnfα isolated from cLP of WT and *Selenow* KO mice at day 8 post DSS (WT = 6 mice; *Selenow* KO = 9). Data indicate mean ± SEM. (**B**,**D**,**F**) Unpaired non-parametric Mann–Whitney test. ** *p* < 0.01, n.s., not significant.

We assessed several inflammatory markers in these mice following DSS administration. Immunoblotting analysis revealed a decreased trend in pro-Il- 1β with a concomitant increase in mature Il-1β in the colonic lysates of *Selenow* KO mice on day 8 post DSS, compared to their WT counterparts (Figure 2A–C). Moreover, as in IBD patients, we found a significantly increased colonic expression of Tnfα in the *Selenow* KO mice compared to the WT mice at day 8 post DSS treatment (Figure 2D,E). Furthermore, flow cytometric analysis of the colonic lamina propria indicated a higher percentage of Tnfα^+^ macrophages in the *Selenow* KO mice compared to the WT mice observed on day 8 post DSS treatment, even though there were no differences in the total percentage of macrophages between the two genotypes post DSS treatment (Figure 3A–D). It has been reported that TNFα produced by Cx3Cr1 macrophages are involved in the pathogenesis of IBD [52]. Along these lines, we observed a greater percentage of Cx3Cr1^+^ Tnfα^+^ macrophages in the *Selenow* KO mice compared to the WT mice at day 8 post DSS treatment (Figure 3E,F). These results further confirm that lack of Selenow exacerbates inflammation, and thereby contributes to impaired resolution of GI inflammation in these mice.

### 3.3. Decreased Intestinal Barrier Integrity in Selenow KO Mice

**Figure 4:**
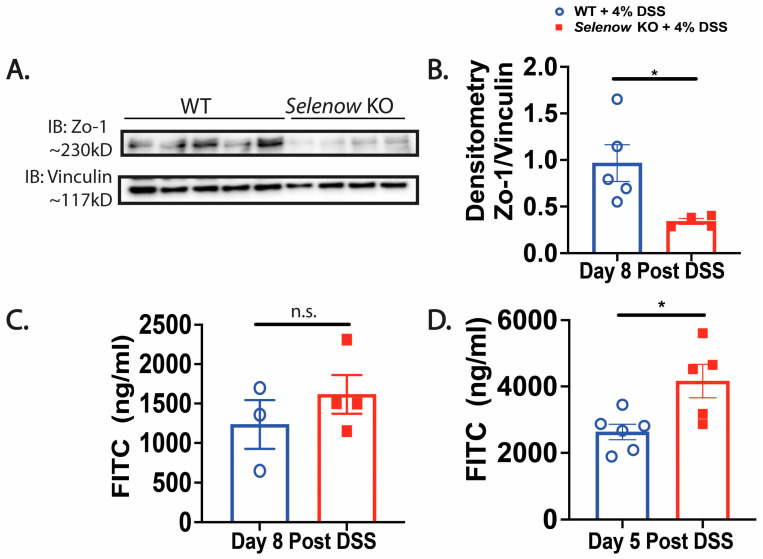
Loss of Selenow exacerbates colonic epithelial barrier disruption following injury. (**A**) Representative immunoblot of Zo-1 and (**B**) quantification of Zo-1 from colonic lysates of WT and *Selenow* KO mice at day 8 post DSS (WT = 5 mice; *Selenow* KO = 4 mice). (**C**) FITC concentration in serum of WT and *Selenow* KO mice at day 8 post DSS (WT = 3 mice; *Selenow* KO = 4). (**D**) FITC concentration in serum of WT and *Selenow* KO mice at day 5 post DSS (WT = 6 mice; *Selenow* KO = 5). Data indicate mean ± SEM. (**B**–**D**) Unpaired non-parametric Mann–Whitney test. * *p* < 0.05, n.s., not significant.

**Figure 5:**
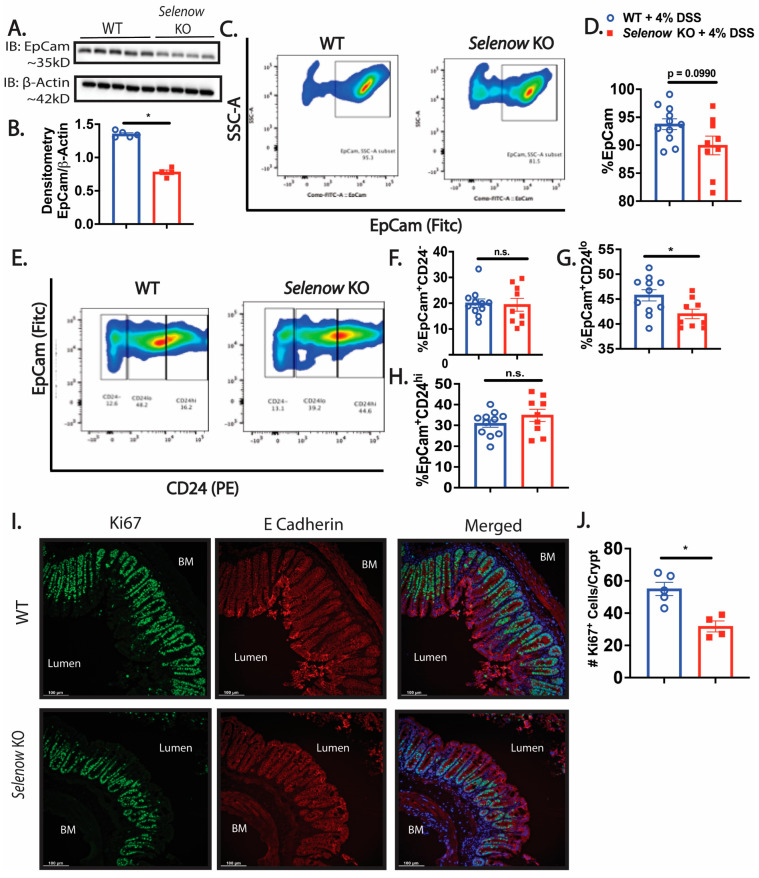
Decreased cycling epithelial cells following injury in *Selenow* KO mice. (**A**) Representative immunoblot of EpCam and (**B**) quantification of EpCam from colonic lysates of WT and *Selenow* KO mice at day 8 post DSS (WT = 5 mice; *Selenow* KO = 4). (**C**) Representative flow plot and (**D**) percentage of epithelial (EpCam^+^) cells isolated from the intestinal epithelium of WT and *Selenow* KO mice at day 8 post DSS (WT = 11 mice; *Selenow* KO = 9). (**E**) Representative flow plot and (**F**–**H**) percentage of EpCam^+^CD24^−^, EpCam^+^CD24^hi^ and cycling cells (EpCam^+^CD24^lo^) isolated from the intestinal epithelium of WT and *Selenow* KO mice at day 8 post DSS (WT = 11 mice; *Selenow* KO = 9). (**I**) Representative immunofluorescent image (20× magnification; scale bar = 100 μm; BM: basement membrane) and (**J**) quantification of the number of Ki67^+^ cells (green: AF488) per crypt (co-stained with E Cadherin (red: AF594) of WT and *Selenow* KO mice at day 8 post DSS (WT = 5 mice; *Selenow* KO = 4). Data indicate mean ± SEM. (**B**,**D**,**F**–**H**,**J**) Unpaired non-parametric Mann–Whitney test. * *p* < 0.05, n.s., not significant.

We investigated the impact of Selenow on the epithelial barrier and observed that in the colonic lysates collected on day 8 post DSS, expression of the barrier protein, Zo-1 was decreased in the *Selenow* KO mice when compared to their WT counterparts (Figure 4A,B). To get a quantitative measure of the changes in barrier integrity, we examined the serum levels of FITC-dextran following oral gavage. While fluorescence spectroscopic measurements indicated an increased trend in serum FITC levels in *Selenow* KO mice at day 8 post DSS (Figure 4C), significantly increased serum FITC levels were observed in the *Selenow* KO mice compared to the WT mice at day 5 post DSS (Figure 4D). Furthermore, the decrease in Zo-1 expression and increased barrier permeability were accompanied by a decrease in EpCam expression in whole colonic lysates (Figure 5A,B) as well as a decreased trend in Epcam^+^ intestinal epithelial cells (Figure 5C,D). Since CD24 expression is used to delineate cycling cells and CD24^lo^ cells are typically enriched in intestinal stem cells [53], we investigated the expression of CD24 in EpCam^+^ intestinal epithelial cells. While there were no differences in EpCam^+^CD24^−^ and EpCam^+^CD24^hi^ cells between *Selenow* KO and WT mice, we found a significant decrease in EpCam^+^CD24^lo^ cycling cells in the *Selenow* KO mice compared to the WT mice (Figure 5E,H). Furthermore, immunofluorescence staining of colonic sections revealed a decrease in the Ki67^+^ cells per crypt in the *Selenow* KO mice compared to the WT mice (Figure 5I,J). These data suggest that Selenow may regulate the intestinal epithelial barrier repair through the control of intestinal stem cell component.

### 3.4. Decreased Yap1 Expression in Selenow KO Mice

**Figure 6:**
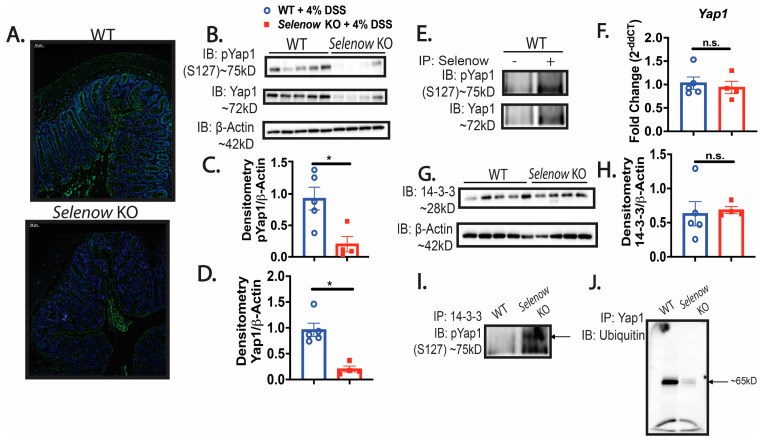
Decreased Yap1 expression in DSS-treated *Selenow* KO mice. (**A**) Representative immunofluorescence images of Yap1(green: AF488) co-stained with DAPI (blue) in WT and *Selenow* KO mice at day 8 post DSS (20× magnification; scale bar = 50 μm). (**B**) Representative immunoblot of pYap1 (S127) and Yap1 and (**C**,**D**) quantification of pYap1 and Yap1 from colonic lysates of WT and *Selenow* KO mice at day 8 post DSS (WT = 5 mice; *Selenow* KO = 4). (**E**) Immunoprecipitation of Selenow with representative immunoblot of pYap1 and Yap1 from colonic lysates of control WT mice. (**F**) Yap1 mRNA expression in colon of WT and *Selenow* KO mice at day 8 post DSS (WT = 5 mice; *Selenow* KO = 4). (**G**) Representative immunoblot of 14-3-3 and (**H**) quantification of 14-3-3 from colonic lysates of WT and *Selenow* KO mice at day 8 post DSS (WT = 5 mice; *Selenow* KO = 4). (**I**) Immunoprecipitation of 14-3-3 with representative immunoblot of pYap1 (S127) from colonic lysates of WT and *Selenow* KO mice at day 8 post DSS (arrow shows pYap1). (**J**) Immunoprecipitation of Yap1 with representative immunoblot of Ubiquitin from colonic lysates of WT and *Selenow* KO mice at day 8 post DSS. Data indicate mean ± SEM. (**C**,**D**,**G**) Unpaired non-parametric Mann–Whitney test. * *p* < 0.05, n.s., not significant.

We investigated whether the compromised barrier integrity could be due to a modulation in Yap1 activation in the *Selenow* KO mice. Immunofluorescence analysis of the tissue sections revealed a lower expression of Yap1 in the *Selenow* KO mice compared to WT mice (Figure 6A). This was further confirmed in the colonic lysates using western immunoblotting, which demonstrated a significant decrease in both phosphorylated Yap1 (S127) and Yap1 expression in *Selenow* KO mice (Figure 6B–D). Furthermore, co-immunoprecipitation (Co-IP) of Selenow and Yap1 clearly confirmed their interaction in the colonic lysates (Figure 6E). Since we observed a decrease in Yap1 in the *Selenow* KO colonic lysates, we explored if Selenow could be regulating the expression of Yap1 mRNA. Real time PCR revealed that there was no difference in the Yap1 mRNA expression between the WT and *Selenow* KO (Figure 6F). Since Yap1 can be sequestered in the cytoplasm by 14-3-3, further examination of whether or not there was a difference in the expression of 14-3-3 suggested no differences between the WT and *Selenow* KO mice (Figure 6G,H). However, IP for 14-3-3 followed by IB for phosphorylated Yap1 (S127), suggested an interaction between 14-3-3 and phosphorylated Yap1 (S127) in the *Selenow* KO colonic lysates, which was clearly absent in the WT lysates (Figure 6I). To examine if the decrease in the Yap1 expression observed in *Selenow* KO mice was related to degradation of Yap1, we analyzed the ubiquitination of Yap1 in the colonic lysates. Ubiquitination of Yap1 appeared to be lower in the *Selenow* KO lysates compared to their WT counterparts (Figure 6J). Overall, our data suggest that Selenow may regulate Yap1 activation, by modulating its interaction with proteins such as the 14-3-3 proteins, ensuring efficient repair and regeneration of the intestinal epithelium.

### 3.5. Decreased Egfr Expression in Mice Lacking Selenow

**Figure 7:**
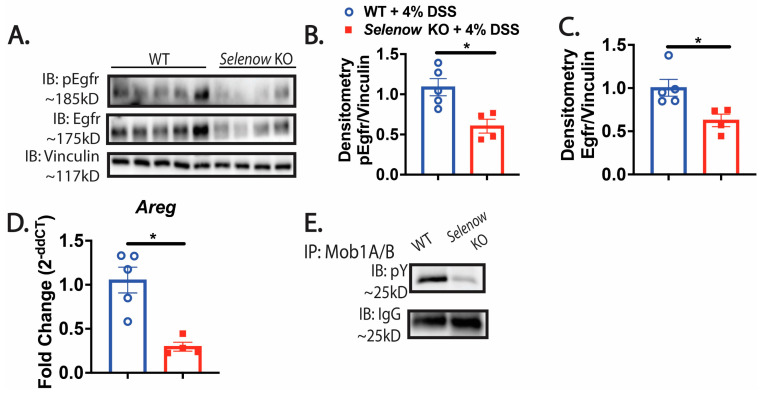
*Selenow* KO mice have decreased Egfr expression following injury. (**A**) Representative immunoblot of pEgfr (Y1068) and Egfr. (**B**,**C**) Quantification of pEgfr (Y1068) and Egfr from colonic lysates of WT and *Selenow* KO mice at day 8 post DSS (WT = 5 mice; *Selenow* KO = 4). (**D**) Areg mRNA expression in colon of WT and *Selenow* KO mice at day 8 post DSS (WT = 5 mice; *Selenow* KO = 4). (**E**) Immunoprecipitation of Mob1A/B with representative immunoblot of phospho-tyrosine (pY) from colonic lysates of WT and *Selenow* KO mice at day 8 post DSS. Data indicate mean ± SEM. (**B**–**D**) Unpaired non-parametric Mann–Whitney test. * *p* < 0.05.

Previous studies in prostate and breast cancer cells demonstrated that Selenow modulates the expression of Egfr by preventing its degradation [40]. In addition, Egfr is intricately involved in GI repair [7,14]. Here, we tested the hypothesis that the difference in epithelial barrier integrity was also related to the regulation of Egfr by Selenow and that lack of Selenow decreased Egfr expression. To address this, we examined the colonic expression of Egfr in WT and *Selenow* KO mice by immunoblotting. Western immunoblotting revealed a clear decrease in Egfr expression in the *Selenow* KO mice compared to the WT mice (Figure 7A–C). Furthermore, DSS treatment of *Selenow* KO mice also led to a significant decrease in the mRNA of *Areg*, a ligand of Egfr, on day 8 post DSS (Figure 7D). Since Areg is a downstream target of Yap1 activation and Egfr can activate Yap1 through the inactivation of Mob1A/B by increasing its tyrosine phosphorylation, we investigated this interaction further. A decrease in Mob1A/B tyrosine phosphorylation (pY) was seen in *Selenow* KO colonic lysates (Figure 7E). Taken together, our data suggests that Selenow modulates Egfr expression and facilitates the cross talk of Egfr with Yap1 to plausibly drive epithelial repair.

### 3.6. Greater Disruption of the Selenow KO Organoids with Yap1 Inhibition

**Figure 8:**
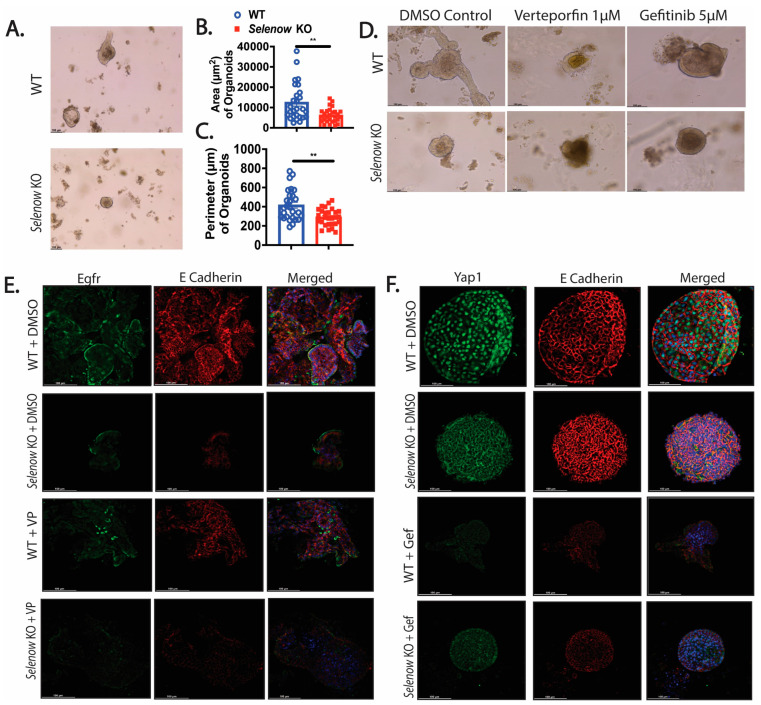
Inhibition of Yap1 and Egfr disrupts organoid morphology. (**A**) Representative image of WT and *Selenow* KO colonoids and (**B**,**C**) quantification of the area and perimeter of the WT and *Selenow* KO colonoids at day 7 of culture (WT = 27 colonoids; *Selenow* KO = 28 colonoids). (**D**) Representative image of WT and *Selenow* KO colonoids at day 9 of culture with 2 days (from day 7 to day 9) of treatment with DMSO, the Yap1 inhibitor, verteporfin (VP: 1 μM), or Egfr inhibitor, gefitinib (Gef: 5 μM). (**E**) Representative immunofluorescence images of Egfr (green: AF488) and E Cadherin (red: AF594) in WT and *Selenow* KO colonoids treated with DMSO or VP (40× magnification; scale bar = 100 μm). (**F**) Representative immunofluorescence images of Egfr (green: AF488) and E Cadherin (red: AF594) in WT and *Selenow* KO colonoids treated with DMSO or Gef (40× magnification; scale bar = 100 μm). (Data indicate mean ± SEM. (**B**,**C**) Unpaired non-parametric Mann–Whitney test. ** *p* < 0.01.

To further explore the underpinnings of the regulation of Yap1 and Egfr by Selenow, colonic intestinal epithelial crypts from both WT and *Selenow* KO mice were used to generate colonic organoids (colonoids). We found that the colonoids generated from WT crypts had a greater area and perimeter than the colonoids generated from *Selenow* KO crypts at day 7 of culture (Figure 8A–C). Microscopic analysis of colonoid morphology indicated that the WT colonoids produced more lobed colonoids compared to the *Selenow* KO colonoids that were unbranched in nature (Figure 8D). Treatment with verteporfin, a Yap1 inhibitor, for 2 days resulted in morphological differences, where both WT and *Selenow* KO treated organoids were disrupted, compared to the vehicle control. However, *Selenow* KO verteporfin-treated organoids appeared to be more disrupted than the WT verteporfin-treated organoids (Figure 8D). Treatment of the WT and *Selenow* KO colonoids with gefitinib, an Egfr inhibitor, also disrupted the morphology when compared to the vehicle-treated controls, though no apparent differences were noted between the gefitinib treated WT and *Selenow* KO colonoids (Figure 8D). To understand if there is a crosstalk between Yap1 and Egfr, Egfr expression in the verteporfin-treated colonoids and Yap1 expression in the gefitinib treated colonoids were examined. We observed that the Egfr expression was lower in the *Selenow* KO DMSO treated organoids compared to the WT DMSO colonoids and that there was a further decrease when the *Selenow* KO colonoids were treated with verteporfin (Figure 8E). Furthermore, Yap1 expression was decreased in the *Selenow* KO DMSO treated colonoids (compared to the WT DMSO-treated colonoids), which was further decreased upon treatment with gefitinib (Figure 8F). Overall, these results suggests that in the context of Selenow, Yap1 appears to be important in maintaining the structure of the epithelium and is key to wound healing and resolution, through crosstalk between Egfr and Yap1, which needs to be further examined in detail.

## 4. Discussion

Previous studies reported the beneficial role of selenium and selenoproteins during IBD and colitis-associated cancer, where the importance of selenoproteins in T cells, macrophages, and intestinal epithelial cells were demonstrated [26,27,28,29,30,39,54]. Our results confirm the expression of *SELENOW* in the normal human intestine and report a decreased expression in inflamed colonic tissues compared to non-inflamed tissue of UC patients. Furthermore, our findings suggest that Selenow plays a key role in the modulation of inflammation and subsequent repair of the intestinal epithelium in an experimental murine model of colitis. Our data indicate the involvement of Selenow in the regeneration of the epithelium following injury, where it mediates the regulation of Yap1 and Egfr activation.

Tumor necrosis factor alpha (Tnfα) suppression has been the target for the treatment of IBD, as it is increased locally (intestinal mucosa) and systemically (blood) in IBD patients [4,55]. However, patients may be unresponsive to the treatments, depending on the type of TNFα inhibitor used, leading to disease relapse [56]. Our studies show that the *Selenow* KO mice have a higher percentage of Tnfα^+^ macrophages in the colonic lamina propria, which further complements Selenow-dependent inverse correlation in Ifnγ^+^Th1 cells in *Selenow* KO mice [39]. Together, these studies provide further evidence of the possible significance of Selenow in regulating inflammation through diverse mechanisms. It is possible that the increase in Tnfα may be driven by NF-κB, especially as it was reported by Huang et al. that the p65 subunit of NF-κB was increased in Th1 cells lacking Selenow expression, which is reminiscent of macrophages lacking the expression of selenoproteins [39,57,58]. Therefore, it is plausible that just the lack of Selenow promotes the activation of NF-κB and Tnfα production leading to exacerbated inflammation, accompanied by inefficient and/or untimely resolution.

The ability of the intestine to be repaired is a key factor for remission in IBD. A greater disruption of the intestinal epithelial barrier in the *Selenow* KO mice is accompanied by a decrease in the percentage of cycling cells (EpCam^+^CD24^lo^) as well as a decrease in both Yap1 and Egfr proteins. Pro-inflammatory cytokines, including Tnfα, are involved in the regulation of tight junction proteins and can also lead to the apoptosis of the intestinal epithelial cells [9,12,13,59]. Therefore, it is possible that the observed difference in Zo-1 could be partially mediated by the greater percentage of Tnfα in the *Selenow* KO mice. In breast and prostate epithelial cells, lack of Selenow result in the arrest of the cell cycle in the G0/G1 phase via Egfr signaling [38]. More importantly, Selenow prevents the degradation of Egfr [40]. Along these lines, the decrease in Egfr in *Selenow* KO colonic lysates may be due to an increase in Egfr degradation. It is clear from our results that Selenow interacts with Yap1 and that the lack of Selenow leads to decreased activation of Yap1 since pYap1 is sequestered in the cytoplasm by 14-3-3 (Figure 6E,J). In fact, Jeon et al. reported that the translocation of the Yap1 coactivator TAZ is decreased when Selenow is knocked down in a skeletal muscle cell line, as it is being sequestered in the cytoplasm by 14-3-3 [60]. Therefore, it is possible that the mice experience impaired repair of the epithelium due to the inactivation of Yap1. These data imply that Selenow may directly or indirectly activate Yap1 to enable nuclear translocation. Surprisingly, we found that the ubiquitination on Yap1 is lower in *Selenow* KO mice, which could be due to the fact that the Yap1 expression is already decreased in the *Selenow* KO mice. Our data highlights the possibilities of a feed forward loop from Yap1 to Egfr since we observe a decrease in *Areg* (Figure 7D). In fact, Yap1 increases Areg expression, thus activating Egfr in a ligand-dependent manner [23,24]. Additionally, it is also possible that there is a convergence of Egfr and Yap1 signaling that is possibly mediated by Mob1, supported by the fact that the *Selenow* KO colonic lysates have a lower phosphorylated Mob1A/B compared to the WT lysates (Figure 7E). Ando et al. recently reported in head and neck squamous cell carcinoma that Egfr could activate Yap1 by phosphorylating Mob1, which further dephosphorylates Lats1/2, thereby inactivating Lats1/2 that leads to the activation of Yap1 [22].

To further understand these interactions, studies using ex vivo colonoid cultures were conducted. It is surprising that without any treatment, the *Selenow* KO derived organoids are significantly smaller than those from WT (Figure 8A), which could be due to a decrease in Yap1 in these organoids (Figure 8E). Furthermore, in line with our studies, Bailey et al. reported that Yap1 is essential for the growth of esophageal organoids [61]. Treatments of organoids with verteporfin results in a more disrupted morphology in the *Selenow* KO colonoids compared to the WT colonoids (Figure 8D). While gefitinib treated organoids also has disrupted morphology, there are no apparent differences between WT and *Selenow* KO colonoids. The organoid data demonstrate that a crosstalk between Egfr and Yap1 may also occur in the context of Selenow, as observed by the lower expression of Egfr in DMSO and verteporfin-treated *Selenow* KO colonoids (Figure 8E). Furthermore, the decrease in Yap1 in DMSO and gefitinib-treated *Selenow* KO organoids also supports the notion of a crosstalk (Figure 8F). Surprisingly, Yap1 expression in the gefitinib-treated *Selenow* KO colonoids is higher than the gefitinib treated WT organoids, which could be a result of other compensatory mechanisms that may lead to a higher Yap1 expression in the organoids. Based on the obvious morphological difference when Yap1 is inhibited, it is possible that Yap1 may play a more crucial role in the context of Selenow. One can speculate that the feedforward loop from Yap1 to Egfr may be more important, versus the activation of Yap1 by Egfr. Further studies are needed to confirm such conclusions.

Our current study involves the usage of only male mice. Future studies will include the use of female mice to increase the generalization and impact that is observed in a Selenow deficient setting. Since DSS results in obliteration of the intestinal epithelium, other models of IBD that are more relevant to humans should be used to confirm the current findings. While we demonstrate that lack of Selenow leads to increased macrophage producing Tnfα and inflammation, our studies did not investigate the effect that the lack of Selenow has on other immune cell types such as T cells. Therefore, future studies should address the impact of Selenow deficiency on the various subsets of T cells, especially Th17 cells which have been implicated in the pathogenesis of IBD. Furthermore, we used a whole body knock out of Selenow. The generation and use of cell type specific Selenow knock out mice under Cre drivers for epithelial cells and macrophages would be helpful in delineating further the effect of Selenow in these cell types, while maintaining Selenow levels in other cell types (a project that is currently being undertaken in our laboratory). Previous studies from our laboratory established that selenium supplementation promotes the resolution of inflammation by upregulating PPARγ and its downstream targets [62]. This may be another mechanism through which Selenow exerts its effects and as such future studies geared at understanding the effect of Selenow on PPARγ activation is being undertaken in our laboratory. Nevertheless, our findings are novel in that we not only demonstrate the anti-inflammatory effect of Selenow, but we also provide evidence that Selenow is involved in repairing and regeneration of the intestinal epithelial barrier, implicating Se supplementation and increased Selenow as target therapies of IBD.

## 5. Conclusions

The current study demonstrate that Selenow is involved in the amelioration of colitogenic symptoms in a model of acute colitis. Not only did the loss of Selenow result in increased weight-loss and shortened colons, but also an increase in Tnfα producing macrophages, disrupted epithelial barrier, and a reduction in cycling cells were observed. These findings, along with the data reporting the regulation of Yap1 and Egfr, implicate Selenow in the regeneration of the epithelium following injury. As previously mentioned, dampening of inflammation using immunosuppressants is one of the main therapies against IBD. These data further support the notion that new therapies, involving dietary Se and/or strategies to increase the expression of Selenow could also assist in intestinal repair following inflammation to promote resolution and remission.

## Data Availability

The data are contained within the article.
